# Cytology–Biopsy Concordance in High-Risk Human Papillomavirus–Positive Women with Abnormal Cytology Findings: Menopause-Stratified Analysis

**DOI:** 10.3390/medicina62040631

**Published:** 2026-03-26

**Authors:** Isik Sozen, Gozde Sahin, Yuksel Ulu, Dilara Yitiz, Basak Ozge Kayan, Ilkbal Temel Yuksel

**Affiliations:** 1 Department of Gynecologic Oncology, Başakşehir Çam and Sakura City Hospital, Başakşehir Neighborhood, G-434 Street, No: 2L, Istanbul 34480, Turkey; sahin.gozde1983@gmail.com (G.S.); basakozgekayan@yahoo.com (B.O.K.); drilkbaltemel@gmail.com (I.T.Y.); 2 Department of Pathology, Başakşehir Çam and Sakura City Hospital, Istanbul 34480, Turkey; y.filizulu@gmail.com (Y.U.); yitizd@gmail.com (D.Y.)

**Keywords:** cervical cytology, human papillomavirus, colposcopy, biopsy, concordance, atypical squamous cells of undetermined significance, low-grade squamous intraepithelial lesion, atypical squamous cells—cannot exclude high-grade squamous intraepithelial lesion, high-grade squamous intraepithelial lesion, cervical intraepithelial neoplasia, menopausal status

## Abstract

*Background and Objectives:* In women who are positive for high-risk human papillomavirus (hrHPV), abnormal cytology necessitates colposcopy and biopsy; however, cytology–histology concordance is variable and may differ by menopausal status. This study aimed to evaluate the concordance between cytologic findings and biopsy results in hrHPV-positive women with abnormal Pap tests and to compare outcomes by menopausal status. *Materials and Methods:* This retrospective, single-center study included 904 hrHPV-positive women with abnormal cytology who underwent colposcopy. Cytology findings [atypical squamous cells of undetermined significance (ASC-US), low-grade squamous intraepithelial lesion (LSIL), atypical squamous cells—cannot exclude high-grade squamous intraepithelial lesion (ASC-H), and high-grade squamous intraepithelial lesion (HSIL)] were compared with histological findings [normal, cervical intraepithelial neoplasia (CIN)-1, -2, -3]. Menopausal status was stratified as premenopausal (<48 years) and postmenopausal (≥48 years). Rates of cyto-histologic concordance, overestimation, and underestimation were calculated. *Results:* The predominant cytological result was ASC-US (61.7%), followed by LSIL (25.3%), whereas histologically, CIN was most common (66.5%; CIN-1: 42.8%, CIN-2: 11.5%, CIN-3: 12.2%). Cytology–biopsy concordance was 50.7%, with overestimation in 35.4% and underestimation in 13.9%. Overestimation was highest in ASC-US (43.9%) and ASC-H (37%), while underestimation was most frequently seen in LSIL cases (27.1%). HSIL cytology showed the highest agreement (85.7%). Conversely, LSIL cytology showed higher cyto-histologic concordance in postmenopausal women, whereas ASC-H and HSIL cytologies were more commonly overestimated in comparison to premenopausal women. Using ASC-H/HSIL as the positive cytology threshold for CIN-2+ detection, sensitivity was 41.1% and specificity was 95.8% [positive predictive value (PPV) 75.2%, negative predictive value (NPV) 84.0%; overall accuracy 82.9%]. The sensitivity and NPV were higher in postmenopausal women than in premenopausal women (50.0% vs. 39.9%; 91.3% vs. 82.3%, respectively). *Conclusions:* Cytology–histology concordance is moderate among women who are hrHPV-positive with abnormal cytology, characterized by notable underestimation in low-grade cytology and strong predictive value in HSIL cases. Menopausal status influences the outcomes; in postmenopausal women, high-grade lesions are less frequent, and diagnostic accuracy for detecting CIN-2+ is higher. These findings highlight the need for age- and menopause-sensitive diagnostic approaches.

## 1. Introduction

Cervical cancer remains a major global health problem, ranking as the fourth most common cancer in women worldwide with approximately 660,000 new cases and 350,000 deaths recorded in 2022 [1]. The development of cervical cancer is strongly linked to persistent infection with high-risk human papillomavirus (hrHPV)—nearly all cases of cervical carcinoma are associated with oncogenic HPV strains [2]. The Papanicolaou (Pap) smear cytology test, in particular, has been an effective tool for identifying abnormal cervical cells at a pre-invasive stage, enabling timely treatment and prevention of cancer progression [3]. In recent years, HPV deoxyribonucleic acid (DNA) testing has been incorporated into cervical screening to improve sensitivity. HPV DNA testing combined with cytology has further enhanced detection of high-grade cervical intraepithelial neoplasia, contributing to additional reductions in cervical cancer rates [4].

However, the agreement between cytology results and histopathological findings on biopsy is not perfect. Cervical cytology can yield false-negative or false-positive results due to factors such as inadequate sampling, fixation/preparation artifacts, or subjective interpretation variability [5]. As a result, an abnormal Pap smear is not always confirmed by a corresponding lesion on biopsy, and vice versa. Studies have reported a wide range of cyto-histological concordance rates: in some analyses, only about 58% of cases showed matching cytology and biopsy results, whereas other studies have found overall concordance exceeding 80% [6,7,8]. The remaining discordant cases represent instances where cytology over-called a lesion that was not substantiated histologically or under-called a significant lesion that biopsy later identified. Notably, one would expect that women who are HPV-positive and Pap smear-positive (both tests indicating risk) have a higher pre-test probability of harboring significant cervical neoplasia. Surprisingly, there is a paucity of published data focusing specifically on this double-positive subgroup in terms of cytology-biopsy correlation.

Recent evidence indicates that cervical intraepithelial neoplasia (CIN)-2+ risk among HPV-positive women varies substantially by HPV genotype and cytology severity, supporting risk-based approaches that incorporate genotyping beyond HPV16/18 [9,10]. Accordingly, the Enduring Consensus guidelines consider extended HPV genotyping acceptable to guide clinical management of a positive HPV test result and provide genotype-informed triage recommendations [9]. Nevertheless, cytology remains an imperfect triage tool in HPV-based screening, and alternative or adjunctive approaches—including p16/Ki-67 dual-stain testing, extended genotyping platforms, and DNA methylation assays—are actively being evaluated [11]. In parallel with advances in secondary prevention, prophylactic HPV vaccination continues to be the cornerstone of primary prevention [12]. Moreover, therapeutic HPV vaccines targeting viral oncoproteins are under active investigation for persistent infection and HPV-related intraepithelial lesions [13].

Furthermore, patient factors such as menopausal status may influence the performance of cytology and the likelihood of cyto-histologic concordance. Postmenopausal women pose particular diagnostic challenges in cervical screening. After menopause, the transformation zone of the cervix often recedes into the endocervical canal, which can make it difficult to adequately visualize and sample during colposcopy examination [14]. At the same time, estrogen deficiency leads to atrophic changes in the cervical epithelium that can mimic high-grade dysplasia on a cellular level, increasing the chance of false-positive cytology results in older women [15,16]. Consistent with these mechanisms, studies have noted that women over approximately 45 years of age are more prone to cytological “over-calling”—for example, a high-grade Pap result that is not confirmed as high-grade disease on histology—and, conversely, are less likely to have their cytology underestimate a lesion, compared to younger women [17]. This uncertainty can lead to dilemmas in clinical decision-making and, in some cases, unnecessary procedures (e.g., unwarranted loop excisions) in older women.

Given the gaps in knowledge outlined above, this study aimed to evaluate the concordance between cytologic findings and biopsy results in patients who test positive for hrHPV and have abnormal Pap smears, with a particular focus on differences between premenopausal and postmenopausal women.

## 2. Materials and Methods

This study was designed as a single-center, observational, retrospective analysis. It was conducted at the Gynecologic Oncology Clinic of Başakşehir Çam and Sakura City Hospital. The study period covered patients who underwent colposcopy between May 2020 and May 2024. Ethical approval was obtained from the Başakşehir Çam and Sakura City Hospital Ethics Committee (Date: 12 February 2025, Approval No: 45). The study adhered to the principles of the Declaration of Helsinki. Due to its retrospective design, the requirement for informed consent was waived by the ethics committee.

### 2.1. Study Population

**Figure 1:**
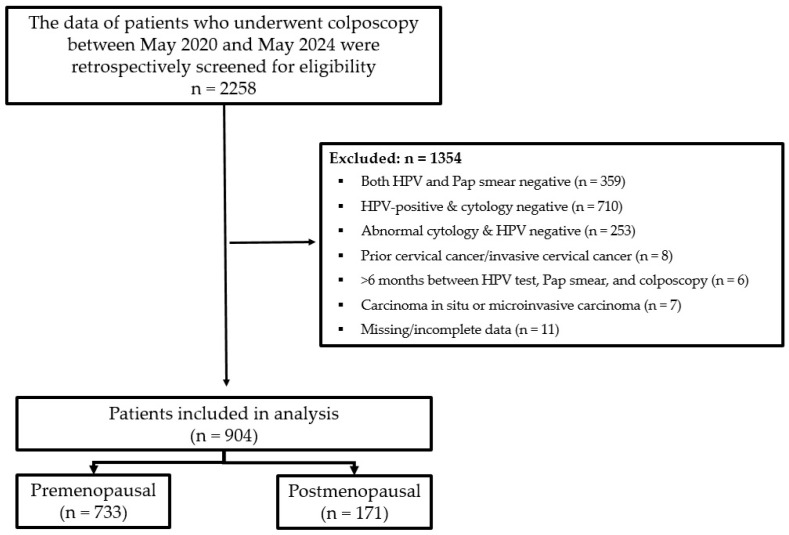
Flowchart of the study population.

A total of 2258 patients who underwent colposcopy during the study period were retrospectively screened. Patients were included in the study if they were ≥18 years old, had positive HPV and Pap smear results, underwent colposcopic evaluation within the study period, and had complete clinical and pathological data available. Patients were excluded if they had only HPV positivity or only Pap smear positivity, had a prior diagnosis of cervical cancer or invasive cervical carcinoma, had an interval longer than six months between HPV testing, Pap smear, and colposcopy, had histopathological findings consistent with carcinoma in situ or microinvasive carcinoma, or had missing or incomplete medical records. After applying the exclusion criteria, the final analysis included 904 patients (Figure 1).

Demographic characteristics (age, menopausal status), clinical history, HPV test results, cytology findings, and histopathology reports were extracted from hospital electronic records. Menopausal status was stratified as premenopausal (<48 years) and postmenopausal (≥48 years). This cutoff value was selected because population-based data or other studies from Türkiye report that the mean/median age at natural menopause is approximately 47–48 years [18,19,20,21], which is earlier than the 50–52 years commonly reported in Western populations [22,23,24].

### 2.2. HPV Genotyping

HPV genotyping is a DNA-based laboratory test to determine whether a person is infected with HPV and, if so, which type (e.g., high-risk 16, 18). This allows for a more accurate assessment of the risk level for cervical cancer and precancerous lesions. The test is usually performed on a cervical swab sample taken from the cervix with a thin brush during a gynecological examination. The sample is placed in a special transport solution and sent to the molecular diagnostic laboratory. In the laboratory, after viral DNA extraction, HPV DNA is amplified using mostly real-time or multiplex polymerase chain reaction (PCR)-based kits, and the presence of genotypes (high/low-risk types) is reported by automated analysis systems. The resulting genotype profile is evaluated together with colposcopy, cytology (Pap smear), and clinical findings to plan the follow-up interval and treatment strategy. HPV genotyping was conducted in the institutional molecular diagnostics laboratory using the Roche Cobas^®^ 4800 diagnostic platform and the Cobas^®^ 4800 HPV Amplification/Detection Kit (Roche Diagnostics GmbH, Mannheim, Germany). The assay identifies 14 high-risk HPV types, including individual detection of HPV16 and HPV18 and pooled detection of 12 additional high-risk HPV types (31, 33, 35, 39, 45, 51, 52, 56, 58, 59, 66, and 68). The test was performed strictly according to the manufacturer’s instructions. The cobas assay includes a β-globin internal cellular control to verify specimen adequacy and to monitor the analytical process. Each run included appropriate positive and negative controls, and runs with invalid results were repeated according to laboratory standard operating procedures. The supplier/service organization (Roche Diagnostics Turkey Inc, İstanbul, Türkiye) maintains a quality management system certified to ISO 9001:2015 standards for in vitro diagnostic devices (TÜV SÜD, Istanbul, Turkey; certificate registration number: 24 3 712925179 [25]; valid from 23 February 2024 to 22 February 2027). Positivity in more than one reporting channel (e.g., HPV16 plus other hrHPV) was recorded. For statistical analysis, patients were categorized hierarchically into three mutually exclusive groups: HPV16-positive (with or without co-positivity), HPV18-positive without HPV16 (with or without co-positivity), and other hrHPV only (negative for HPV16/18). Because the “other hrHPV” channel is pooled, individual non-16/18 genotypes were not analyzed separately.

### 2.3. Cervical Cytology Evaluation

Cervical cytology specimens were interpreted in routine clinical practice and reported according to the 2014 Bethesda System. Cytology results for the present study were retrieved from the final signed-out pathology reports. Given the retrospective design, no additional study-specific blinded slide re-review was performed, and interobserver agreement statistics were not available.

Atypical squamous cells of undetermined significance (ASC-US): This category includes squamous cells that show nuclear and/or cytoplasmic abnormalities exceeding benign/reactive changes but that are insufficient, either in degree or quality, to fulfill the criteria for a definitive squamous intraepithelial lesion.

Low-grade squamous intraepithelial lesion (LSIL): LSIL corresponds mainly to transient HPV-related changes and cervical intraepithelial neoplasia grade 1 (CIN-1), characterized by koilocytosis and mild nuclear atypia involving the superficial and intermediate cell layers.

High-grade squamous intraepithelial lesion (HSIL): HSIL encompasses cytologic changes associated with CIN-2 and CIN-3, showing marked nuclear enlargement, hyperchromasia, irregular chromatin, and increased nuclear-to-cytoplasmic ratio, often involving the parabasal and intermediate cell layers.

Atypical squamous cells—cannot exclude HSIL (ASC-H): This category is used when squamous cells display worrisome atypia suggestive of, but quantitatively or qualitatively insufficient for, a definitive diagnosis of HSIL; it indicates a possible high-grade lesion and mandates further diagnostic evaluation such as colposcopy and biopsy.

### 2.4. Histopathological Evaluation

Cytology results were additionally dichotomized into a low-risk group, comprising ASC-US and LSIL, and a high-risk group, comprising ASC-H and HSIL.

Colposcopic examinations were performed using a binocular colposcope equipped with a green filter (Olympus OCS 500). Following irrigation of the cervix with normal saline, the transformation zone was evaluated under green filter illumination to better visualize atypical vascular patterns. Subsequently, 3% acetic acid was applied, and prior to obtaining punch biopsies, acetowhite lesions, abnormal vascular patterns, and other suspicious areas were carefully documented. The biopsy specimens consisted of small fragments of cervical tissue.

Tissue samples were fixed in neutral buffered formalin, embedded in paraffin, and sectioned at 4 μm thickness. Two serial sections from each block were stained with hematoxylin and eosin for routine light microscopic examination. Histopathology was classified according to WHO criteria; however, for the purposes of this study, outcomes were restricted to benign/CIN-1/CIN-2/CIN-3, and carcinoma in situ or microinvasive carcinoma were excluded.

### 2.5. Definition of Cyto-Histologic Correlation

Cytology–biopsy correlation was defined as the degree of concordance between the cytological interpretation and the histopathological diagnosis of colposcopy-directed biopsy specimens. For analytic purposes, cytology–histology correlation was categorized as overestimated, concordant, or underestimated (Table 1). Concordance was defined as ASC-US or LSIL cytology with CIN-1 on biopsy, and as ASC-H or HSIL cytology with CIN-2/3 on biopsy. Overestimation was defined as ASC-US or LSIL cytology with benign/non-neoplastic histology, or as ASC-H or HSIL cytology with benign or CIN-1 histology. Underestimation was defined as ASC-US or LSIL cytology with CIN-2/3 on biopsy. Underestimation was not defined for ASC-H/HSIL within this analytic framework because lesions more severe than CIN-3 were not included in the analysis. This categorization follows a lesion-grade correlation approach consistent with cytology–histology correlation (CHC) frameworks used in quality assurance practice [6].

### 2.6. Statistical Analysis

Data analysis was conducted with IBM SPSS Statistics for Windows, version 20.0 (IBM Corp., Armonk, NY, USA). The normality of the data distribution was evaluated using the Shapiro–Wilk test. Descriptive statistics were presented as means ± standard deviations (SD) or medians with interquartile ranges (IQR) for continuous variables, and as frequencies and percentages for categorical variables. Group comparisons between premenopausal and postmenopausal patients were conducted using the chi-square test or Fisher’s exact test for categorical variables, and independent-samples *t*-test or Mann–Whitney U test for continuous variables, as appropriate. Receiver operating characteristic (ROC) analysis was used to assess the diagnostic performance of binary cytology (ASC-H/HSIL vs. ASC-US/LSIL) in predicting biopsy-confirmed CIN-2+ lesions, and AUC values with corresponding 95% confidence intervals (CIs) were presented. A *p*-value of <0.05 was considered statistically significant for all analyses.

## 3. Results

The mean age of the study participants was 38.6 ± 10.2 years, with the vast majority in the premenopausal stage. HPV typing revealed that HPV16 was the most prevalent genotype (37.9%), followed by HPV18 (9.4%) and other hrHPV types (52.7%). Cytological findings demonstrated that the majority of patients presented with ASC-US (61.7%), while LSIL and HSIL were identified in 25.3% and 7.0% of cases, respectively. Histopathological evaluation showed that 33.5% of patients had non-malignant lesions, whereas 66.5% were diagnosed with CIN of varying severity. Among positive cases, CIN-1 was the most common diagnosis (42.8%), followed by CIN-2 (11.6%) and CIN-3 (12.2%) (Table 2).

When stratified according to menopausal status, significant differences emerged between premenopausal and postmenopausal women. Distribution of HPV genotypes did not differ significantly between the groups (*p* = 0.347). Cytological patterns were also similar, with ASC-US being the predominant finding in both groups (60.3% in premenopausal *vs*. 67.8% in postmenopausal women, *p* = 0.165). However, histopathological outcomes showed notable differences: postmenopausal women had lower rates of CIN-2/3 (15.2% vs. 25.6%, *p* = 0.011) (Table 2).

### 3.1. Correlation of Cytological Findings with Histological Results

When cytological results were analyzed in relation to histological outcomes, distinct patterns were observed. Among patients with non-malignant histology, the majority had ASC-US cytology (80.9%), while LSIL (15.1%), ASC-H (3.0%), and HSIL (1.0%) were less common. In CIN-1 lesions, ASC-US remained the predominant cytology (64.3%), followed by LSIL (31.3%), whereas ASC-H (2.8%) and HSIL (1.6%) were rare. For CIN-2, 43.3% of cases were classified as ASC-US, 32.7% as LSIL, 13.5% as ASC-H, and 10.5% as HSIL. In CIN-3, cytological categorization shifted toward higher grades, with HSIL accounting for 39.0% of cases, LSIL for 25.5%, ASC-H for 18.2%, and ASC-US for 17.3% (Table 3).

### 3.2. Findings of Cyto-Histologic Correlation

When cytological interpretations were compared with histopathological diagnoses, 50.7% of cases were concordant, whereas 35.4% were overestimated and 13.9% were underestimated. Overestimation was most frequent in ASC-US cytology (43.9%). LSIL showed moderate concordance (52.8%), with 20.1% overestimated and 27.1% underestimated. ASC-H cytology was concordant with CIN ≥ 2 in 63.0% of cases, while 37.0% were overestimated. By contrast, 85.7% of patients diagnosed with HSIL cytology had histologically confirmed CIN ≥ 2, while the remaining 14.3% were overestimated. In subgroup analyses by menopausal status, distributions among premenopausal women were consistent with the overall cohort. Compared with premenopausal women, postmenopausal women with ASC-US cytology exhibited a higher rate of overestimation and a lower rate of underestimation. While LSIL cytology demonstrated higher concordance in postmenopausal women, ASC-H and HSIL cytologies were more frequently overestimated compared with premenopausal women (Table 4).

From the histology perspective, concordance differed markedly by lesion severity (Table 5). Among biopsy-confirmed CIN-1 lesions, cytology was concordant (ASC-US/LSIL) in 95.6% of cases, while 4.4% were overestimated (ASC-H/HSIL). In contrast, among biopsy-confirmed CIN ≥ 2 lesions, cytology was concordant (ASC-H/HSIL) in 41.1% of cases, whereas 58.9% were underestimated (ASC-US/LSIL).

### 3.3. Diagnostic Performance of Cytology Findings

The diagnostic performance of dichotomized cytology for identifying histology-confirmed CIN ≥ 2 is summarized in Table 6. For this analysis, ASC-H/HSIL was considered the positive group and ASC-US/LSIL the negative group, using colposcopy-directed biopsy as the reference standard. The overall diagnostic performance of cytology revealed sensitivity of 41.1%, specificity of 95.8%, and accuracy of 82.9%. The premenopausal group showed lower sensitivity compared to the postmenopausal group, while specificity rates were comparable. Additionally, the postmenopausal group demonstrated lower PPV but higher NPV, whereas the overall accuracy was greater in postmenopausal women (Table 6).

## 4. Discussion

The key findings of our study are as follows. First, in this cohort of 904 hrHPV-positive women with abnormal cytology, overall cytology–histology concordance was moderate, with substantial discordance driven mainly by overestimation and, to a lesser extent, underestimation. Using an ASC-H threshold (ASC-H/HSIL vs. ASC-US/LSIL) to predict biopsy-confirmed CIN2+, cytology demonstrated low sensitivity but high specificity, with a high overall accuracy. Second, concordance and misclassification patterns differed markedly by cytology subtype: HSIL showed the highest concordance with CIN2/3, whereas low-grade categories—particularly LSIL—contributed disproportionately to underestimation of high-grade lesions. Third, menopausal status influenced these patterns; compared with premenopausal women, postmenopausal women more often showed cytologic overestimation (especially in ASC-US, ASC-H, and HSIL), while underestimation tended to be less frequent, and diagnostic performance metrics shifted in a direction consistent with lower CIN2+ prevalence in this group.

The reported concordance between Pap smear interpretations and cervical biopsy results varies widely (approximately 40% to 95%) depending on the population and definitions used [26,27,28,29]. Our cohort focuses specifically on women who were double-positive (hrHPV-positive with abnormal cytology), a clinically important subgroup because concurrent positivity is associated with higher underlying CIN risk [10]. Despite this higher-risk profile, the overall concordance remained only moderate, highlighting that cyto-histologic discordance persists even in populations enriched for disease. Our results showed that cytology missed a significant proportion of important lesions. In fact, in our cohort of hrHPV-positive women, approximately 60% of all high-grade histological lesions (CIN2+) were reported as ASC-US or LSIL on Pap smear cytology. This is comparable to a previous report indicating that 73.4% of women with high-grade CIN had low-grade cytology [30]. This kind of underestimation contributes to the lower sensitivity of the Pap test. A comprehensive review found that the sensitivity of Pap for CIN2+ is approximately 51% [31], meaning that about half of the precancerous lesions may be missed in a single scan—this figure is high. This is consistent with the ~41% sensitivity we observed for CIN2+ detection. On the other hand, the high specificity of Pap smear is also important; our data shows that in studies, most women with HSIL Pap results had high-grade lesions on excisional evaluation [26,32]. Other studies have reported similar PPV. In a 2022 study by Miki et al., among women with HSIL smears, the rate of CIN2+ on biopsy was 87% in younger patients [33].

This finding has important implications for triage strategies that rely mainly on cytology severity. In our cohort, 126 of 214 CIN2+ cases (58.9%) occurred in women categorized as ASC-US/LSIL, corresponding to a CIN2+ prevalence of 16.0% (126/787) within the ASC-US/LSIL subgroup. Therefore, in hrHPV-positive women, lower-grade abnormal cytology should not be interpreted as reassuring, and management pathways should avoid depending on cytology grade alone to exclude significant disease. In addition, the agreement between cytology and colposcopy-guided biopsy is affected by variability in both sampling and interpretation. As neither modality alone ensures full diagnostic accuracy, dependence on a single reference standard has inherent limitations. Accordingly, current American Society for Colposcopy and Cervical Pathology (ASCCP) guidelines recommend an integrated approach incorporating cytology, HPV testing, and histopathology to guide clinical management [34]. Adjunctive biomarkers may help address this underdetection by enriching for transforming infections among hrHPV-positive women with ASC-US/LSIL. In particular, p16-based approaches (e.g., p16/Ki-67 dual stain on cytology specimens) have been proposed as triage tools in HPV-positive populations and may improve identification of underlying CIN2+/CIN3+ compared with cytology alone, while preserving specificity [35]. In addition, p16 immunohistochemistry can improve diagnostic reproducibility in selected histopathologic scenarios (e.g., morphologically equivocal CIN2), helping to distinguish true high-grade disease from benign mimics [36]. This may also be relevant in postmenopausal women, in whom atrophic epithelial changes can contribute both to cytologic overcalling and to discordance. Future studies in double-positive cohorts should evaluate whether combining biomarker-based triage with HPV genotyping can reduce the proportion of CIN2+ lesions categorized as ASC-US/LSIL, while also limiting unnecessary procedures in groups prone to overestimation.

When considering differences by cytological subtype, we found distinct concordance and discordance patterns. Specifically, approximately 44% of ASC-US cytology cases and 20% of LSIL cytology cases were identified as histologically overestimated, while underestimation rates were 11.5% for ASC-US and 27% for LSIL. Recent hrHPV-positive cohorts show that a clinically meaningful proportion of ASC-US cases harbor CIN, including CIN2+ [37,38]. Kim et al., in their study, revealed HSIL in 14.6% of patients with ASC-US cytology through histopathological examination [39]. Low-grade cytological changes such as LSIL have a more modest concordance of around 60–70% in various studies [26,29]. However, it has been reported that the PPV of Pap test findings positively correlates with lesion severity, approximately 38% for LSIL, 62% for HSIL, and 100% for invasive carcinoma [40]. HSIL cytology has strong positive predictive value for significant disease, and histopathological confirmation has been reported in approximately 80% or more of HSIL cases [26]. This is to be expected, as high-grade lesions are less likely to be missed or misinterpreted on cytology or biopsy, whereas low-grade changes may be subtle or prone to sampling variance [41,42].

Recent large cohorts confirm that the prevalence of CIN2/3 among women with ASC-H/HSIL varies by HPV genotype and age, and that older/postmenopausal women can show higher rates of histologic downgrading/over-interpretation [43,44]. In our cohort, postmenopausal women had lower rates of CIN2/3 than premenopausal women, and the distribution of discordance shifted toward overestimation, particularly for ASC-US and ASC-H, while underestimation tended to be less common. Choi et al. observed that 27% of postmenopausal patients with high-grade cytology (ASC-H/HSIL) had only low-grade histological findings—more than double the rate of such “overestimation” (≈12%) in younger women [30]. Conversely, among those with mild cytological abnormalities, a much higher proportion of premenopausal women had high-grade CIN on biopsy compared to postmenopausal women [30]. ASC-H classification is associated with variability and a relatively high false positive rate, particularly in older women [28]. In 37% of ASC-H cases, cytology results were exaggerated, and these cases were histologically confirmed as low-grade lesions or benign. In the literature, among women with ASC-H cytology, less than 6% of women over 55 years of age had true high-grade lesions, while this rate was approximately 22% in perimenopausal women [45]. In another study on postmenopausal women with ASC-H cytology, Ahn et al. detected histologically confirmed HSIL in 18% of their cases, while the remaining cases had LSIL or negative biopsies [46]. This pattern has also been documented by previous researchers. Gilani et al. reported that more than half (52.5%) of Pap smear abnormalities in postmenopausal women were not confirmed by corresponding biopsy, compared to about one-third (33.6%) in premenopausal women [27]. A large Korean cohort study found that patients with ASC-H over 45 years of age were twice as likely to overestimate the lesion prior to cervical biopsy, while those under 45 were more prone to underestimation [17]. Notably, the study by Miki et al. also showed that the PPV of HSIL dropped to approximately 65% in women over 50 years of age [33], highlighting an age effect that we also observed (there was more HSIL “overestimation” in older women). We observed that approximately 14% of HSIL smears did not have high-grade lesions on biopsy, and such false positives were largely in postmenopausal patients.

The biological and technical reasons behind this cytological-histological discrepancy are multifactorial. In postmenopausal women, there tends to be a regressed transformation zone located higher up in the endocervical canal, making comprehensive sampling difficult during Pap smears or colposcopic biopsies [14,47]. The difficulty in correctly interpreting ASC-H smears in elderly patients is often attributed to atrophic epithelial changes mimicking high-grade dyskaryosis [41,48]. Postmenopausal cervical atrophy can produce cellular features that cytologists may interpret as possible HSIL, leading to a high false positive rate in this group. In contrast, younger women with ASC-H are more likely to have underlying true HSIL/CIN2+; therefore the accurate prediction of ASC-H is higher in this population. These aggregate results demonstrate the need to customize cytological assessment according to age or menopausal status to improve histological agreement and optimize clinical management strategies.

### Limitations of Study

Limitations of this study should be taken into account when interpreting the results. The retrospective, single-center design may introduce selection bias and restrict the generalizability of the findings to other populations or screening settings with different patient profiles and protocols. Furthermore, the study exclusively included women who were positive for both hrHPV and cytology, representing a high-risk subset; thus, the concordance rates observed may not be applicable to HPV-positive women with normal cytology or to lower-risk groups. In addition, detailed colposcopic findings were not consistently available for all patients due to the retrospective nature of the dataset, which prevented stratified analyses evaluating the potential impact of colposcopic abnormalities on the diagnostic accuracy of cytology. Importantly, long-term follow-up data were not available; therefore, we could not evaluate lesion progression or regression over time, and it remains uncertain whether some discordant or apparently “false-positive” cytology results reflect true overcalling, early regression/transient HPV-related abnormalities, or lesions missed due to the known sampling limitations of colposcopy-directed biopsy. In addition, the relatively small sample of postmenopausal women—particularly the low number of postmenopausal CIN2/3 cases—limited statistical power for menopause-stratified comparisons and yielded wider uncertainty around subgroup estimates; therefore, subgroup findings should be interpreted cautiously. Finally, because cytology interpretations were based on finalized clinical reports, we were unable to conduct a dedicated slide re-review and quantify interobserver agreement. Future research should prioritize adequately powered, multicenter and preferably prospective cohorts (or registry-based studies) with larger/oversampled postmenopausal representation, standardized diagnostic protocols, and longitudinal follow-up to strengthen inference on subgroup differences.

## 5. Conclusions

This study underscores that while Pap smear remains central to cervical screening, its concordance with pathology is imperfect, particularly at age extremes. Clinicians should note the high risk of occult high-grade lesions in younger women with minor cytologic abnormalities and false-positive smears in postmenopausal women. A combined approach—integrating cytology, hrHPV testing, and menopausal status-aware judgment—is vital to optimize detection of significant lesions and avoid overtreatment. Our findings advocate individualized screening algorithms and sustained vigilance in interpreting and following abnormal Pap smears across all ages.

## Figures and Tables

**Table 1:** Definitions used for cytology–histology correlation categories (concordant, overestimated, underestimated).

Category	Definition	Example
Concordant	Cytological and histological findings are aligned with respect to lesion grade.	ASC-US/LSIL cytology with CIN-1 on histology ASC-H/HSIL cytology with CIN-2/3 on histology
Overestimated	Cytology suggests a higher-grade abnormality than that confirmed by histopathology.	ASC-US/LSIL cytology with benign/non-neoplastic histology ASC-H/HSIL cytology with benign/CIN-1 histology
Underestimated	Cytology indicates a lower-grade abnormality than that ultimately identified on histological examination.	ASC-US/LSIL cytology with CIN-2/3 on biopsy

Abbreviations: ASC-H, atypical squamous cells-cannot exclude HSIL; ASC-US, atypical squamous cells of undetermined significance; CIN, cervical intraepithelial neoplasia; HSIL, high-grade squamous intraepithelial lesion; LSIL, low-grade squamous intraepithelial lesion.

**Table 2:** Distribution of demographic and clinical findings.

Variables	Overall Population n = 904	Premenopausal n = 733	Postmenopausal n = 171	*p*-Value
Age, years	38.6 ± 10.2	35.2 ± 7.8	53.4 ± 4.3	<0.001
HPV, n (%)				
HPV16	343 (37.9)	281 (38.3)	62 (36.3)	0.347
HPV18	85 (9.4)	73 (10.0)	12 (7.0)	
Others	476 (52.7)	379 (51.7)	97 (56.7)	
Cytology, n (%)				
ASC-US	558 (61.7)	442 (60.3)	116 (67.8)	0.165
LSIL	229 (25.3)	195 (26.6)	34 (19.9)	
ASC-H	54 (6.0)	42 (5.7)	12 (7.0)	
HSIL	63 (7.0)	54 (7.4)	9 (5.3)	
Histopathology, n (%)				
Non-malignant	303 (33.5)	235 (32.1)	68 (39.8)	0.011 *
CIN-1	387 (42.8)	310 (42.3)	77 (45.0)	
CIN-2/3	214 (23.7)	188 (25.6)	26 (15.2)	
CIN-2	104 (11.5)	93 (12.6)	11 (6.4)	0.021 *
CIN-3	110 (12.2)	95 (13.0)	15 (8.8)	

Data are mean ± SD or numbers and column percentages. * *p*-value < 0.05 shows statistical significance. Abbreviations: ASC-H, atypical squamous cells—cannot exclude HSIL; ASC-US, atypical squamous cells of undetermined significance; CIN, cervical intraepithelial neoplasia; HSIL, high-grade squamous intraepithelial lesion; HPV, human papillomavirus; LSIL, low-grade squamous intraepithelial lesion.

**Table 3:** Distribution of cytological findings by histological results.

Cytology		Histology				
		Non-Malignant	CIN-1	CIN-2	CIN-3	CIN ≥ 2
Overall population	ASC-US	245 (80.9)	249 (64.3)	45 (43.3)	19 (17.3)	64 (29.9)
	LSIL	46 (15.1)	121 (31.3)	34 (32.7)	28 (25.5)	62 (29.0)
	ASC-H	9 (3.0)	11 (2.8)	14 (13.5)	20 (18.2)	34 (15.9)
	HSIL	3 (1.0)	6 (1.6)	11 (10.5)	43 (39.0)	54 (25.2)
Premenopausal	ASC-US	186 (79.1)	199 (64.2)	41 (44.1)	16 (16.8)	57 (30.3)
	LSIL	41 (17.4)	98 (31.6)	31 (33.3)	25 (26.3)	56 (29.8)
	ASC-H	6 (2.6)	8 (2.6)	11 (11.8)	17 (17.9)	28 (14.9)
	HSIL	2 (0.9)	5 (1.6)	10 (10.8)	37 (38.9)	47 (25.0)
Postmenopausal	ASC-US	59 (86.8)	50 (64.9)	4 (36.4)	3 (20.0)	7 (26.9)
	LSIL	5 (7.4)	23 (29.9)	3 (27.3)	3 (20.0)	6 (23.1)
	ASC-H	3 (4.4)	3 (3.9)	3 (27.3)	3 (20.0)	6 (23.1)
	HSIL	1 (1.5)	1 (1.3)	1 (9.1)	6 (40.0)	7 (26.9)

Data are numbers and column percentages. Abbreviations: ASC-H, atypical squamous cells—cannot exclude HSIL; ASC-US, atypical squamous cells of undetermined significance; CIN, cervical intraepithelial neoplasia; HSIL, high-grade squamous intraepithelial lesion; LSIL, low-grade squamous intraepithelial lesion.

**Table 4:** Findings of cyto–histology correlation.

Cytology	Overestimated	Concordant	Overestimated
Overall population, n (%)			
ASC-US	245 (43.9)	249 (44.6)	64 (11.5)
LSIL	46 (20.1)	121 (52.8)	62 (27.1)
ASC-H	20 (37.0)	34 (63.0)	0
HSIL	9 (14.3)	54 (85.7)	0
Total	320 (35.4)	458 (50.7)	126 (13.9)
Premenopausal, n (%)			
ASC-US	186 (42.1)	199 (45.0)	57 (12.9)
LSIL	41 (21.0)	98 (50.3)	56 (28.7)
ASC-H	14 (33.3)	28 (66.7)	0
HSIL	7 (13.0)	47 (87.0)	0
Total	248 (33.8)	372 (50.8)	113 (15.4)
Postmenopausal, n (%)			
ASC-US	59 (50.9)	50 (43.1)	7 (6.0)
LSIL	5 (14.7)	23 (67.6)	6 (17.6)
ASC-H	6 (50.0)	6 (50.0)	0
HSIL	2 (22.2)	7 (77.8)	0
Total	72 (42.1)	86 (50.3)	13 (7.6)

Data are numbers and row percentages. Abbreviations: ASC-H, atypical squamous cells—cannot exclude HSIL; ASC-US, atypical squamous cells of undetermined significance; HSIL, high-grade squamous intraepithelial lesion; LSIL, low-grade squamous intraepithelial lesion.

**Table 5:** Biopsy-stratified cyto–histology correlation by lesion severity (CIN-1 vs. CIN ≥ 2).

Histology	Overestimated	Concordant	Underestimated
Overall population, n (%)			
CIN-1, n = 387	17 (4.4)	370 (95.6)	-
CIN ≥ 2, n = 214	-	88 (44.1)	126 (58.9)
Premenopausal, n (%)			
CIN-1, n = 310	13 (4.2)	297 (95.8)	-
CIN ≥ 2, n = 188	-	75 (39.9)	113 (60.1)
Postmenopausal, n (%)			
CIN-1, n = 77	4 (5.2)	73 (94.8)	-
CIN ≥ 2, n = 26	-	13 (50.0)	13 (50.0)

Data are numbers and row percentages. For CIN-1 histology, concordance corresponds to ASC-US/LSIL cytology and overestimation corresponds to ASC-H/HSIL. For CIN ≥ 2 histology, concordance corresponds to ASC-H/HSIL cytology and underestimation corresponds to ASC-US/LSIL. Abbreviations: ASC-H, atypical squamous cells—cannot exclude HSIL; ASC-US, atypical squamous cells of undetermined significance; CIN, cervical intraepithelial neoplasia; HSIL, high-grade squamous intraepithelial lesion; LSIL, low-grade squamous intraepithelial lesion.

**Table 6:** Diagnostic performance of dichotomized cytology (ASC-H/HSIL vs. ASC-US/LSIL) for predicting biopsy-confirmed CIN ≥ 2 lesions.

Cytology	Histology	
	CIN ≥ 2	CIN < 2
Overall population	n = 214	n = 690
ASC-H/HSIL, n (%)	88 (41.1)	29 (4.2)
ASC-US/LSIL, n (%)	126 (58.9)	661 (95.8)
AUC ± SE, % (95% CI)	0.69 ± 0.02 (0.64–0.73)	
Sensitivity, % (95% CI)	41.1 (34.5–48.0)	
Specificity, % (95% CI)	95.8 (94.0–97.2)	
PPV, % (95% CI)	75.2 (67.3–81.8)	
NPV, % (95% CI)	84.0 (82.4–85.5)	
Accurate rate, % (95% CI)	82.9 (80.2–85.3)	
Premenopausal	n = 188	n = 545
ASC-H/HSIL, n (%)	75 (39.9)	21 (3.8)
ASC-US/LSIL, n (%)	113 (59.1)	524 (96.2)
AUC ± SE, % (95% CI)	0.68 ± 0.03 (0.63–0.73)	
Sensitivity, % (95% CI)	39.9 (32.8–47.3)	
Specificity, % (95% CI)	96.2 (94.2–97.6)	
PPV, % (95% CI)	78.1 (69.4–84.9)	
NPV, % (95% CI)	82.3 (80.5–83.9)	
Accurate rate, % (95% CI)	81.7 (78.7–84.5)	
Postmenopausal	n = 26	n = 145
ASC-H/HSIL, n (%)	13 (50.0)	8 (5.5)
ASC-US/LSIL, n (%)	13 (50.0)	137 (94.5)
AUC ± SE, % (95% CI)	0.72 ± 0.06 (0.60–0.85)	
Sensitivity, % (95% CI)	50.0 (29.9–70.1)	
Specificity, % (95% CI)	94.5 (89.4–97.6)	
PPV, % (95% CI)	61.9 (42.8–77.9)	
NPV, % (95% CI)	91.3 (87.8–93.9)	
Accurate rate, % (95% CI)	87.7 (81.8–92.2)	

Data are numbers and column percentages. Reference standard was histology from colposcopy-directed biopsy. CIN2+ was defined as CIN2 or CIN3; ≤CIN1 included benign/non-neoplastic histology and CIN1. True positive (TP) = ASC-H/HSIL with CIN2+; false negative (FN) = ASC-US/LSIL with CIN2+; false positive (FP) = ASC-H/HSIL with ≤CIN1; true negative (TN) = ASC-US/LSIL with ≤CIN1. Denominators: sensitivity = TP/(TP + FN) among CIN2+; specificity = TN/(TN + FP) among ≤CIN1; Positive predictive value (PPV) = TP/(TP + FP) among ASC-H/HSIL; Negative predictive value (NPV) = TN/(TN + FN) among ASC-US/LSIL. Overall accuracy = (TP + TN)/N. Abbreviations: ASC-H, atypical squamous cells—cannot exclude HSIL; ASC-US, atypical squamous cells of undetermined significance; CIN, cervical intraepithelial neoplasia; HSIL, high-grade squamous intraepithelial lesion; LSIL, low-grade squamous intraepithelial lesion.

## Data Availability

The data that support the findings of this study are available on request from the corresponding author due to privacy and ethical reasons.
